# Impact of Vitamin D Replacement on Markers of Glucose Metabolism and Cardio-Metabolic Risk in Women with Former Gestational Diabetes—A Double-Blind, Randomized Controlled Trial

**DOI:** 10.1371/journal.pone.0129017

**Published:** 2015-06-09

**Authors:** Toh Peng Yeow, Shueh Lin Lim, Chee Peng Hor, Amir S. Khir, Wan Nazaimoon Wan Mohamud, Giovanni Pacini

**Affiliations:** 1 Department of Medicine, Penang Medical College, Penang, Malaysia; 2 Steno Diabetes Centre, Gentofte, Denmark; 3 Department of Medicine, Penang General Hospital, Penang, Malaysia; 4 Clinical Research Centre, Seberang Jaya Hospital, Seberang Jaya, Penang, Malaysia; 5 Kepala Batas Hospital, Kepala Batas, Penang, Malaysia; 6 Cardiovascular, Diabetes and Nutrition Research Centre, Institute for Medical Research, Kuala Lumpur, Malaysia; 7 Metabolic Unit, Institute of Biomedical Engineering, National Research Council, Padova, Italy; Indiana University Richard M. Fairbanks School of Public Health, UNITED STATES

## Abstract

Gestational Diabetes Mellitus (GDM) and vitamin D deficiency are related to insulin resistance and impaired beta cell function, with heightened risk for future development of diabetes. We evaluated the impact of vitamin D supplementation on markers of glucose metabolism and cardio metabolic risk in Asian women with former GDM and hypovitaminosis D. In this double blind, randomized controlled trial, 26 participants were randomized to receive either daily 4000 IU vitamin D3 or placebo capsules. 75g Oral Glucose Tolerance Test (OGTT) and biochemistry profiles were performed at baseline and 6 month visits. Mathematical models, using serial glucose, insulin and C peptide measurements from OGTT, were employed to calculate insulin sensitivity and beta cell function. Thirty three (76%) women with former GDM screened had vitamin D level of <50 nmol/L at baseline. Supplementation, when compared with placebo, resulted in increased vitamin D level (+51.1 nmol/L vs 0.2 nmol/L, p<0.001) and increased fasting insulin (+20% vs 18%, p = 0.034). The vitamin D group also demonstrated a 30% improvement in disposition index and an absolute 0.2% (2 mmol/mol) reduction in HbA_1c_. There was no clear change in insulin sensitivity or markers of cardio metabolic risk. This study highlighted high prevalence of vitamin D deficiency among Asian women with former GDM. Six months supplementation with 4000 IU of vitamin D3 safely restored the vitamin D level, improved basal pancreatic beta-cell function and ameliorated the metabolic state. There was no effect on markers of cardio metabolic risk. Further mechanistic studies exploring the role of vitamin D supplementation on glucose homeostasis among different ethnicities may be needed to better inform future recommendations for these women with former GDM at high risk of both hypovitaminosis D and future diabetes.

## Introduction

Gestational diabetes mellitus (GDM) is a state of glucose intolerance occurring during pregnancy and is related to both resistance to peripheral action of insulin and impairment of beta-cell function. Its transient presence during pregnancy alerts to a heightened risk of diabetes in the future. About 10–50% of women with GDM develop diabetes mellitus later on in life [1]. Data from Malaysia found that 50% of GDM women had developed diabetes at an interval of five to seven years post index pregnancy [2]. Vitamin D deficiency has been shown to be associated with insulin resistance and impaired pancreatic function [3, 4]. Vitamin D deficiency is more prevalent in women with GDM and low vitamin D levels correlate with insulin resistance [5–7].

Interventional studies using vitamin D supplement in an attempt to modify glucose metabolism have yielded mixed results. This may be partly due to variable doses of supplementation used, short duration of follow up and inappropriate target group. A very short duration of less than seven days of supplementation may not be sufficient to demonstrate the potential beneficial effects [8–10]. Previous studies suggested that vitamin D can help with early stage of disturbance in glucose handling [11], but is unable to augment insulin secretion in subjects with chronic diabetes and exhausted pancreatic function [9]. Lack of adequate dosing may have also accounted for the failure of many previous studies to demonstrate beneficial effects of vitamin D replacement. Adequate vitamin D supplementation would ideally raise blood 25-hydroxyvitamin D (25(OH)D) levels above 80 nmol/L because diabetes risk is lowest at this vitamin D level [12]. Supplementation with 4000 IU of vitamin D3 per day for six months in a population of South Asian women with proven vitamin D deficiency safely restored the vitamin D level and improved insulin resistance [13].

Very little is known about the relationship between vitamin D status and glucose metabolism in women with former GDM. Most of the works on effect of vitamin D supplementation on GDM examined its effect during pregnancy rather than following delivery [14].

Our study aimed to evaluate the effect of adequate vitamin D supplementation on insulin sensitivity, pancreatic beta-cell function and markers of cardio-metabolic risk in Malaysian women with former GDM and vitamin D insufficiency.

## Materials and Methods

The protocol for this trial and supporting CONSORT checklist are available as supporting information; see S1 CONSORT Checklist and S1 Protocol.

### Study Design and Screening

This was a prospective, randomized, double-blind, placebo-controlled, parallel group trial. Women with documented GDM in the most recent pregnancy, who were between 6 to 48 months post-partum, were identified from the delivery registry of Maternity Unit, Penang General Hospital and antenatal records of community health centers. Potential participants were approached through postal and telephone contacts, pre-screened by telephone interview and invited to attend the screening visit at Clinical Research Centre at Penang Medical College, Penang, Malaysia. The screening and recruitment period was started from 30^th^ June 2011 through to 3^rd^ August 2012.

Women were excluded if they had medical conditions that might increase their risk or interfere with study assessments. These included pregnancy, breastfeeding, intolerance to vitamin D supplementation, drug or alcohol dependence, chronic renal or liver failure, hypercalcaemia, hypocalcaemia or concomitant use of calcium supplementation, anti-tuberculosis treatment or anti-epileptic medications. Women who have developed diabetes by the time of assessment were also excluded on the rationale that those with established disease might not respond as well to vitamin D supplementation and also to avoid confounding effect of hypoglycaemic medications on assessment of glucose metabolism.

After providing written informed consent and being interviewed, participants underwent anthropometric examinations (body mass index (BMI) and waist-to-hip circumference ratio (WHR)), blood sampling for 75 g oral glucose tolerance test (75g-OGTT), HbA_1c_, fasting lipid profile, plasma 25(OH)D, intact parathyroid hormone (iPTH), renal profile, liver profile, bone profile, highly sensitive CRP (hsCRP) and urinary microalbumin-creatinine ratio (MCR). The 75g-OGTT was performed with serial measurements for serum insulin at time 0, 60, 90 and 120 minutes, C-peptide at time 0, 60 and 120 minutes and glucose at time 0, 30, 60, 90 and 120 minutes. Blood samples were processed on the same day for plasma and serum, and aliquots were stored frozen at -20°C until laboratory analyses were performed. Physical activity was quantified using the Paffenbarger Physical Activity Questionnaire [15].

The Elecsys Vitamin D Total assay (Roche Diagnostics GmbH, Sandhofer Strasse 116, D-68305 Mannheim, Germany) was used to assay total vitamin D, including both vitamin D3 and vitamin D2. Inter-assay coefficient of variation for total vitamin D at concentrations of 37.44 nmol/L was less than 8.5%. The third generation Whole PTH (1–84) Specific Immunoradiometric Assay (IRMA) kits (Scantibodies Laboratory, Inc. 9336 Abraham Way, Santee, CA 92071 USA) were employed to assay iPTH. The human insulin and hsCRP ELISA kits (Demeditec Diagnostics, Lise-Meitne-Strt.2, D-24145 Kiel-Wellsee, Germany) were used to measure serum insulin and hsCRP levels. The two-site immunoenzymometric assay kit (ST AIA-PACK C-peptide, TOSOH Corporation, Shiba-koen First Bldg., 3-8-2 Shiba, Minato-ku, Tokyo 105-8623 Japan) were used to assay C-peptide using the TOSOH AIA System Analyzer. Inter-assay coefficient of variation for insulin at 53 μIU/ml was 3.8%, for C-peptide at 1.98 ng/ml and 4.9 ng/ml were 1.8% and 4.9%, for hsCRP at 2.6 ug/ml was 8.4% and for iPTH at 32 pg/ml was 3.1%.

### Randomization and follow up

Women who have plasma 25(OH)D level between 15 and 50 nmol/L without overt diabetes were eligible to proceed to the randomization phase. Overt diabetes was defined as fasting plasma glucose of ≥7 mmol/L and/or 2-hour OGTT of ≥11.1 mmol/L [16]. Women with plasma 25(OH)D of less than 15 nmol/L or those with overt diabetes were excluded from the study and referred to receive active treatment. Eligible women were then randomized to receive either 4000 IU of vitamin D3 (cholecalciferol) per day (four capsules of 1000 IU each) or four capsules of matching placebo per day for six months. Both the active (vitamin D3) and placebo capsules contained 300 mg of soy oil. Offsite randomization was generated by the manufacturer of the vitamin D and placebo capsules (Blackmore Ltd) using nQuery Advisor. Randomization and allocation were fully concealed from the investigators and subjects until after completion of the study. All participants attended every second month for review and collecting new supply. Participants were advised to contact research staff immediately if they suspected a reaction to the supplements or in the event of pregnancy. Serum calcium and albumin were taken at two months to rule out hypervitaminosis. The assessments performed at the screening visit were repeated after six months of supplementation. The study was completed on 1^st^ February 2013 with last visit for the last participant. All the data were hosted at Penang Medical College. The data were de-identified before their use in analyses.

### Insulin sensitivity and pancreatic beta-cell function

Fasting insulin sensitivity was estimated with the Quantitative Insulin Sensitivity Check Index (QUICKI), while the peripheral insulin sensitivity with the Oral Glucose Insulin Sensitivity Index (OGIS) that represents glucose clearance [17, 18]. These indices have been validated toward the glucose clamp and tracer-calculated hepatic sensitivity [19].

Fasting insulin secretion was evaluated from basal insulin and C-peptide concentrations. Total (120 minutes) insulin secretion was evaluated with the area under the curve of C-peptide (AUC_cp_), while the amount of insulin reaching the periphery (post-hepatic) with AUC of insulin (AUC_insulin_). Beta-cell function was assessed with the insulinogenic index calculated at 60 minutes: IGI_60_ = (I_60_-I_basal_)/ (G_60_-G_basal_), and with the ratio of the incremental AUC_cp_ (ΔAUC_cp_) over the incremental AUC_glucose_ (ΔAUC_glucose_), as a global insulinogenic index [20].

A simultaneous measurement of pancreatic beta-cell function, insulin sensitivity and glucose tolerance test (BIGTT) was employed to provide an additional estimate of insulin sensitivity index (BIGTT-S) and acute insulin response (BIGTT-AIR) using serial data from OGTT; the BIGTT indices were shown to highly correlate with intravenous glucose tolerance test (IVGTT)-derived values [21]. Under normal physiology, the tight glucose regulation is maintained by a balance between insulin secretion and insulin sensitivity [22]. To describe this process, here we used the product of post-hepatic beta-cell function (ratio of AUC_insulin_ over AUC_glucose_) and insulin sensitivity (OGIS). This product, in other circumstances called disposition index (DI), interprets glucose tolerance of an individual by relating beta-cell function with whole-body insulin sensitivity[22].

### Sample size and statistical analyses

By using nQuery Advisor version 6.01, based on a trial by von Hurst et al [13], a sample size of 9 will have 95% power to detect a difference in mean for Homeostatic Model Assessment of Insulin Resistance (HOMA%IR) of 0.700 (e.g. the differences between Group 1 mean, μ1, of 0.800 and a Group 2 mean, μ2, of 0.100), assuming that the common standard deviation is 0.384, with a 0.05 two-sided significance level. By allowing a 40% drop-out rate of subjects, the total sample for two arms (placebo and treatment groups) was calculated to be 26 subjects (13 patients for each arm).

All data in this study were analysed according to intention-to-treat protocol. Missing data for subjects who defaulted and withdrew from the study were analysed using listwise deletion technique. All clinical and biochemical continuous variables, except for post-partum duration, were not normally distributed. The non-normally distributed continuous variables were reported in median (25^th^, 75^th^ percentiles) while the only normally distributed continuous variable, post-partum duration, was reported in mean ± standard deviation. Comparison between interventional and placebo groups for baseline socio-demography, clinical histories and characteristics, markers for glucose metabolism, insulin sensitivity, pancreatic beta-cell function and cardio-metabolic risk markers were performed using Mann-Whitney U tests for non-normally distributed continuous variables. Wilcoxon signed rank tests were employed to detect differences in clinical and biochemical markers pre- and post-intervention in each group, while Mann-Whitney U tests were performed to compare these markers across both groups. The two-sided statistical significance level, p-value, was set at 0.05 for all analyses in this study.

### Ethical approval

The study was registered at Malaysian National Medical Research Register (NMRR) (reference number NMRR-10-679-6202) on 5^th^ June 2010 and ClinicalTrials.gov (NCT01992133) on 12^th^ November 2013. At time of publication, the authors became aware of NMRR is yet to be recognized by the WHO International Clinical Trials Registry Platform, hence the delay in registration of this study with ClinicalTrials.gov. The Medical Research and Ethics Committee (MREC), Ministry of Health Malaysia approved the study protocol by 18^th^ October 2010. Written informed consents were obtained from all participants. The authors confirmed that all ongoing and related trials for intervention are registered.

## Results

### Diabetes and Vitamin D status among women with former GDM

**Fig 1:**
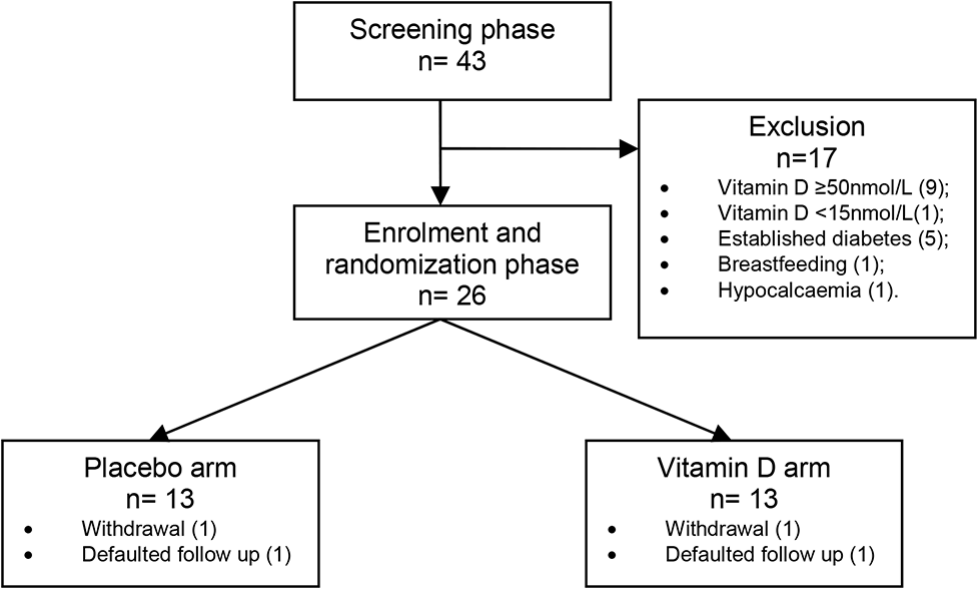
Flow chart of study protocol.

Fig 1 shows the number of participants screened and randomized. A total of 43 women with former GDM were screened for the study at an average of 23 ± 10.7 months post-partum. These were representative of the Malaysian ethnic groups, with 21 (48.8%) Malaysian Malays, 14 (32.6%) Malaysian Chinese and 8 (18.6%) Malaysian Indians. Thirty-three (76%) of these former GDM women had hypovitaminosis (9% had level <20 nmol/L, 67% had level between 20 and 49.9 nmol/L). The median serum vitamin D level among these former GDM women was 40.0 (26.26, 49.67) nmol/L.

By the time of their screening visits, 12 women with former GDM (30.2%) had developed variable degrees of glucose intolerance. These included 1 impaired fasting glucose (IFG), 6 impaired glucose tolerance (IGT) and 5 overt diabetes.

### Randomization and adherence to vitamin D supplementation

**Fig 2:**
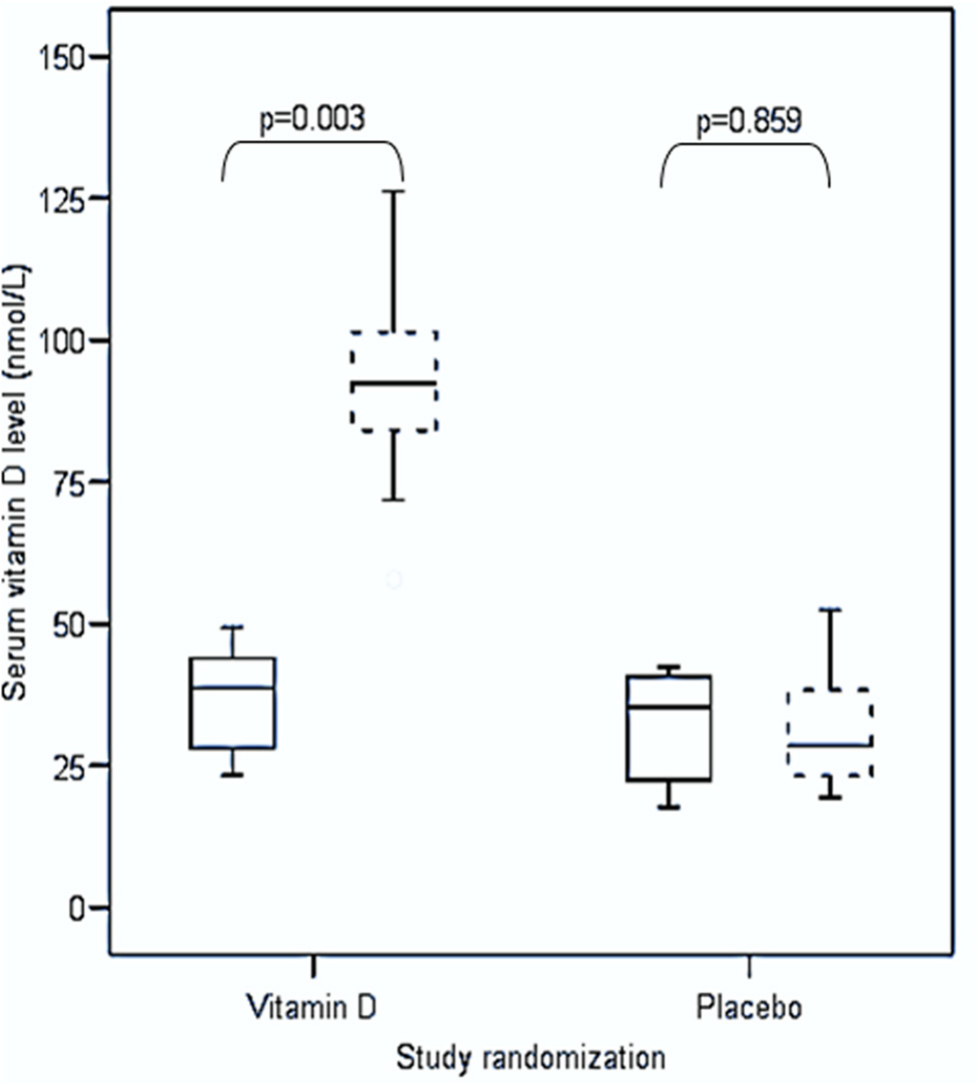
Differences in median serum vitamin D levels among women with former GDM in both groups. Boxplot with complete line = Pre-study serum vitamin D level. Boxplot with breaking line = Post-study serum vitamin D level.

Seventeen women screened were excluded from the randomization phase. Four women dropped out of study (one became pregnant, one developed allergic reaction to bovine capsule and two did not return for final visit due to busy family commitment). Eleven women in each group completed the study (Fig 1). The vitamin D and placebo groups did not differ at baseline with respect to socio-demographics, physical characteristics and laboratory measures, except for serum iPTH and serum total cholesterol (Table 1). The average vitamin D capsule adherence over the 6 months follow-up period, reported by pill count, was 88.2% in the vitamin D group and 86.7% in the placebo group. The median serum 25(OH)D concentrations increased significantly in the vitamin D-supplemented group following 6 months of supplementation while the levels remained unchanged in the placebo group (Fig 2). There was no evidence of hypervitaminosis, and serum calcium after 2 months and 6 months of supplements remained normal for all subjects. One participant developed allergic rash to the bovine capsule which resolved fully on stopping the supplement. No other adverse events were reported.

**Table 1:** Baseline demographics, clinical characteristics and laboratory measures of women with former GDM in both interventional and placebo groups at screening visit.

	Vitamin D group (n = 13)	Placebo group (n = 13)	
	Median (25^th^, 75^th^ percentiles)	Median (25^th^, 75^th^ percentiles)	p-value between groups
Age (years)	36 (32, 38)	35 (30, 40)	0.661
Post-partum duration (days)	731 (460, 951)	496 (406, 858)	0.369
Paffenbarger physical activity (Kcal/ week)	1997 (857, 14562)	2000 (1346, 8508)	0.898
Blood pressure			
Systolic (mmHg)	123 (108, 131)	115 (112, 126)	0.537
Diastolic (mmHg)	81 (70, 84)	72 (68, 83)	0.554
Pulse rate (bpm)	77 (67, 88)	76 (71, 80)	0.521
Body mass index (kg/ m^2^)	26.5 (21.9, 30.7)	28.8 (25.6, 40.0)	0.397
Waist-to-hip circumference ratio	0.89 (0.80, 0.97)	0.89 (0.84, 0.93)	0.778
Serum vitamin D level (nmol/L)	35.6 (25.60, 43.95)	35.1 (21.63, 40.75)	0.249
Intact parathyroid hormone (pg/mL)	25.9 (21.90, 33.40)	16.5 (8.55, 23.20)	0.007
Fasting glucose (mmol/L)	4.8 (4.35, 5.15)	4.8 (4.50, 4.90)	0.817
2-hour glucose (mmol/L)	6.7 (5.45, 8.30)	5.7 (4.60, 6.95)	0.238
HbA_1c_ (%); (mmol/mol)	5.4 (5.20, 5.75); 36 (33, 39)	5.3 (5.00, 5.90); 34 (31, 41)	0.554
Fasting insulin (pmol/L)	79.2 (53.10, 111.00)	81.0 (56.70, 116.40)	0.880
Fasting C-peptide (ng/ml)	0.45 (0.255, 1.133)	0.61 (0.313, 0.708)	0.755
Highly sensitive C-reactive protein (μg/mL)	2.4 (1.45, 7.60)	2.2 (0.90, 7.50)	0.504
Urinary microalbumin-creatinine ratio (mg/mmol)	1.0 (0.53, 2.70)	1.0 (0.70, 1.30)	0.702
Serum total cholesterol (mmol/L)	5.6 (4.65, 6.15)	4.7 (4.20, 5.45)	0.025
Serum low-density lipoprotein (mmol/L)	3.9 (3.05, 4.20)	3.0 (2.60, 3.80)	0.052
Serum high-density lipoprotein (mmol/L)	1.2 (1.05, 1.50)	1.2 (1.00, 1.30)	0.725
Serum triglyceride (mmol/L)	1.1 (0.80, 1.30)	1.0 (0.85, 1.20)	0.837
Serum triglyceride to high-density lipoprotein ratio	0.90 (0.558, 1.039)	0.91 (0.615, 1.154)	0.772

### Effect of vitamin D supplementation on glucose metabolism

**Fig 3:**
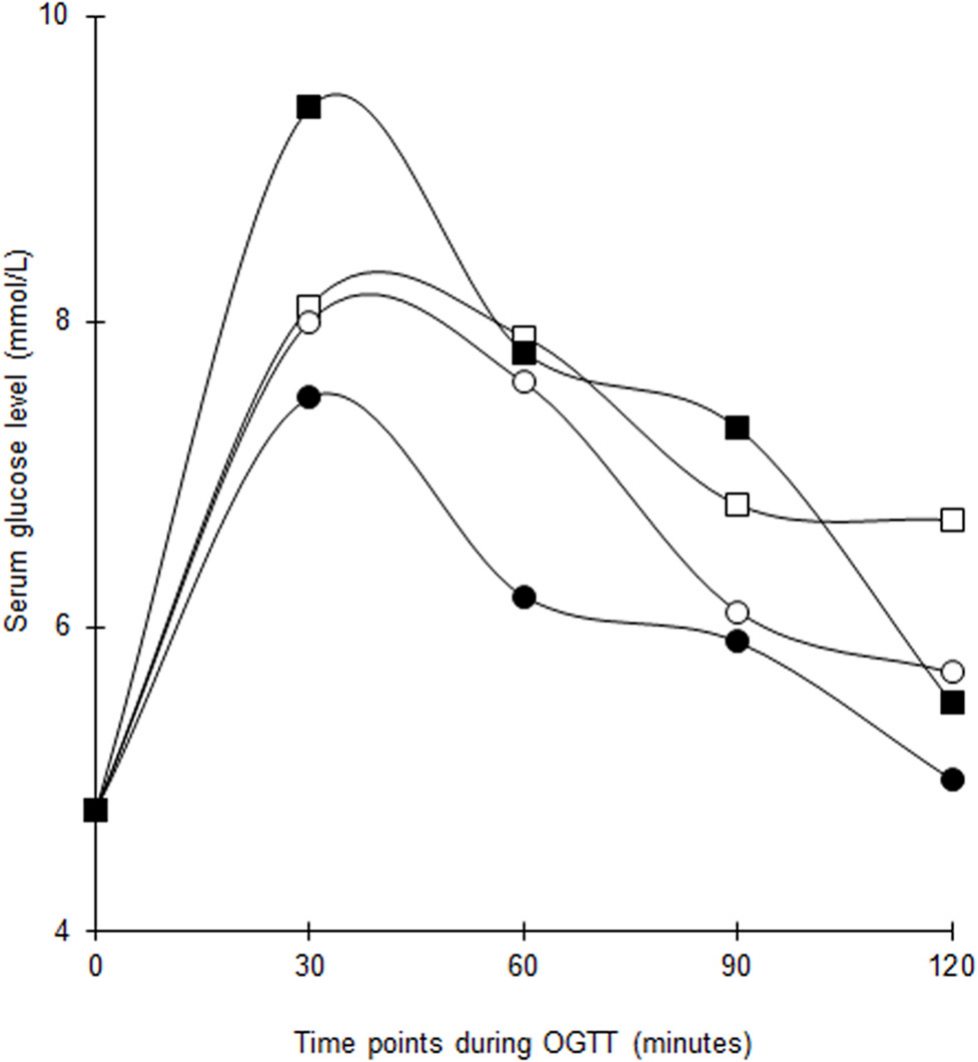
Changes in median serum glucose levels throughout 2-hour OGTT at baseline and post intervention among women with former GDM in both groups. White squares = Vitamin D group (baseline). Black squares = Vitamin D group (post intervention). White circles = Placebo group (baseline). Black circles = Placebo group (post intervention). The baseline differences of median serum glucose levels between vitamin D-supplemented group and placebo group at 0 (p = 0.817), 30 (p = 0.512), 60 (p = 0.383), 90 (p = 0.700) and 120 (p = 0.238) minutes were not statistically significant.

**Fig 4:**
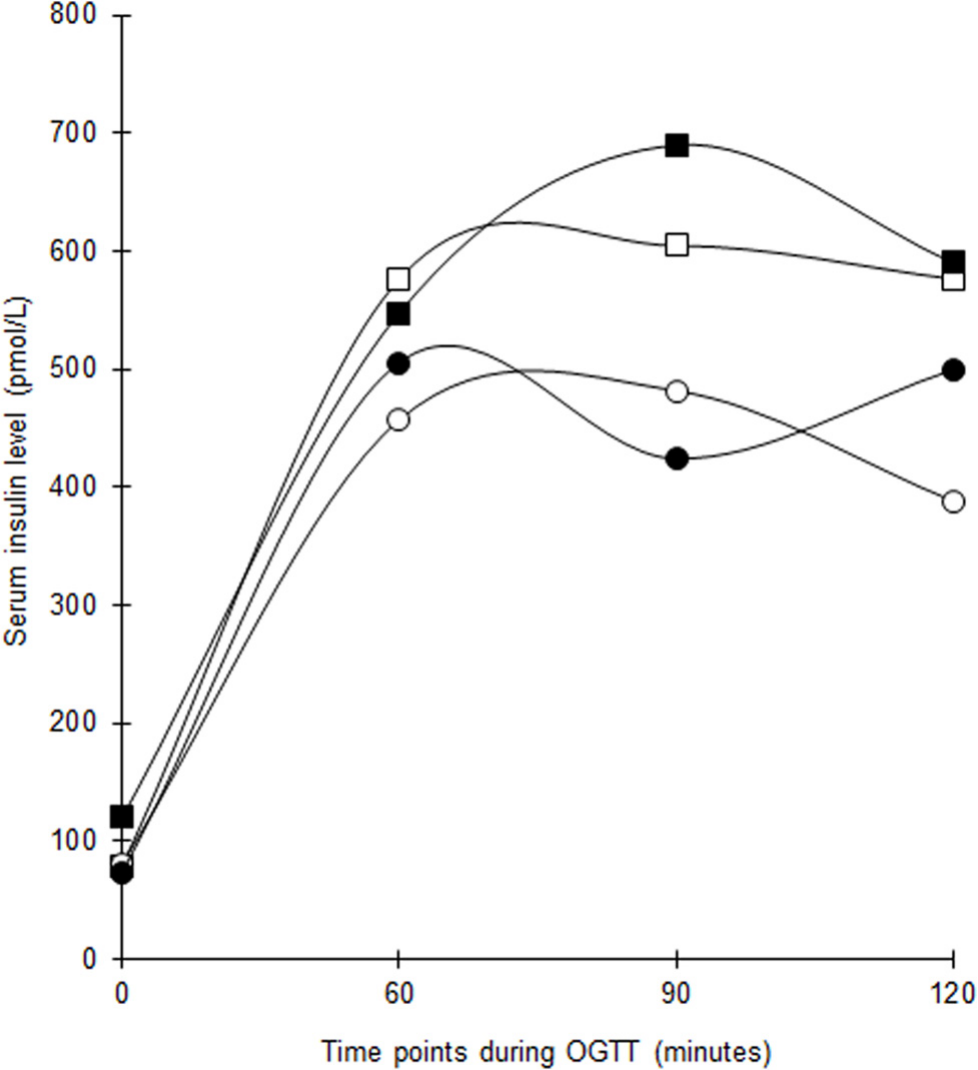
Changes in median serum insulin levels throughout 2-hour OGTT at baseline and post intervention among women with former GDM in both groups. White squares = Vitamin D group (baseline). Black squares = Vitamin D group (post intervention). White circles = Placebo group (baseline). Black circles = Placebo group (post intervention).The baseline differences of median serum insulin levels between vitamin D-supplemented group and placebo group at 0 (p = 0.878), 60 (p = 0.457), 90 (p = 0.489) and 120 (p = 0.112) minutes were not statistically significant.

The serial changes in median serum glucose and insulin levels during OGTT are shown in Figs 3 and 4. The median glucose in the vitamin D group decreased significantly at 2-hour but the changes at the other time points were not significant. There was no difference in the total AUC_glucose_ within and between groups following supplementation. The HbA_1c_ dropped by 2 mmol/mol (0.2%) in both groups but with greater statistical significance in the vitamin D group (Table 2).

**Table 2:** Changes in vitamin D level, glucose and markers of insulin sensitivity and pancreatic beta-cell functions among women with former GDM in both interventional and placebo groups before and after intervention.

	Vitamin D group (n = 13)		Placebo group (n = 13)		
	Median (25^th^, 75^th^ percentiles)	p-value difference within group	Median (25^th^, 75^th^ percentiles)	p-value difference within group	p-value changes(E-B) between groups
**Serum vitamin D level (nmol/L)**					
Baseline (B)	35.6 (25.60, 43.95)	0.003	35.1 (21.63, 40.75)	0.859	
Endpoint (E)	92.4 (79.00, 102.34)		28.5 (20.87, 42.43)		
Change (E-B)	51.1 (39.86, 76.08)		-0.2 (-10.18, 11.83)		<0.001
**Intact parathyroid hormone (pg/mL)**					
Baseline (B)	25.9 (21.90, 33.40)	0.213	16.5 (8.55, 23.20)	0.656	
Endpoint (E)	20.1 (16.00, 29.00)		17.0 (10.10, 22.50)		
Change (E-B)	-5.1 (-12.50, 1.40)		2.30 (-3.90, 6.90)		0.123
**HbA**_**1C**_ **level and glucose metabolism during OGTT**					
**HbA**_**1c**_ **(mmol/mol); (%)**					
Baseline (B)	36 (33, 39); 5.4 (5.20, 5.75)	0.009	34 (31, 41); 5.3 (5.00, 5.90)	0.057	
Endpoint (E)	33 (30, 36); 5.2 (4.90, 5.40)		31 (30, 40); 5.0 (4.90, 5.80)		
Change (E-B)	-2 (-3, -1); -0.2 (-0.30, -0.10)		-2 (-4, 0); -0.2 (-0.40, 0)		0.847
**Fasting glucose (mmol/L)**					
Baseline (B)	4.8 (4.35, 5.15)	0.674	4.8 (4.50, 4.90)	0.211	
Endpoint (E)	4.8 (4.50, 5.10)		4.8 (4.30, 5.30)		
Change (E-B)	0 (-0.20, 0)		0.1 (-0.20, 0.50)		0.270
**30-minute glucose (mmol/L)**					
Baseline (B)	8.1 (7.50, 9.95)	0.646	8.0 (7.15, 9.10)	0.342	1.000
Endpoint (E)	9.4 (7.20, 10.30)		7.5 (6.80, 7.90)		
Change (E-B)	-0.4 (-2.40, 1.70)		-0.1 (-1.93, 0.48)		
**2-hour glucose (mmol/L)**					
Baseline (B)	6.7 (5.45, 8.30)	0.009	5.7 (4.60, 6.95)	0.657	
Endpoint (E)	5.5 (5.00, 7.10)		5.0 (4.50, 7.90)		
Change (E-B)	-2.6 (-1.50, -0.40)		0.4 (-1.20, 1.00)		0.061
**AUC**_**glucose**_ **(mmol/L)**					
Baseline (B)	871.5 (773.25, 1095.00)	0.386	849.0 (702.75, 942.00)	0.182	
Endpoint (E)	871.5 (768.00, 1027.50)		727.5 (655.50, 969.00)		
Change (E-B)	-28.5 (-199.50, 70.5)		-56.6 (-130.50, 54.00)		1.00
**ΔAUC**_**glucose**_ **(mmol/L)**					
Baseline (B)	283.5 (210.00, 496.50)	0.423	264.0 (140.25, 379.5)	0.062	
Endpoint (E)	286.5 (216.00, 441.00)		157.5 (79.50, 333.00)		
Change (E-B)	-28.5 (-228, 70.5)		-60 (-129.00, 12.00)		0.606
**Insulin secretion**					
**Fasting insulin (pmol/L)**					
Baseline (B)	79.2 (53.10, 111.0)	0.003	81.0 (56.70, 116.4)	0.003	
Endpoint (E)	120.0 (73.80, 127.20)		73.8 (46.20, 102.00)		
Change (E-B)	15.6 (-13.80, 51.00)		-14.4 (-46.20, 0)		0.034
**Fasting C-peptide (ng/mL)**					
Baseline (B)	0.4 (0.26, 1.13)	0.013	0.6 (0.31, 0.71)	0.021	
Endpoint (E)	1.1 (0.57, 1.61)		0.8 (0.57, 1.22)		
Change (E-B)	0.4 (0.03, 0.54)		0.3 (-0.09, 0.48)		0.365
**AUC**_**insulin**_ **(pmol/L)**					
Baseline (B)	60660 (47931.0, 69399.0)	0.155	46296 (39441.0, 69579.0)	0.477	
Endpoint (E)	66612 (46380.0, 146160.0)		55044 (43242.0, 76134.0)		
Change (E-B)	17376 (-8574, 41514)		3894 (-10242, 17524)		0.365
**AUC**_**cp**_ **(ng/mL)**					
Baseline (B)	240 (157.0, 317.0)	0.004	268 (138.5, 392.5)	0.006	
Endpoint (E)	397 (295.0, 456.0)		380 (371.0, 449.0)		
Change (E-B)	157 (79.0, 210.0)		134 (86.0, 269.0)		1.00
**ΔAUC**_**insulin**_ **(pmol/L)**					
Baseline (B)	48282 (36846.0, 54345.0)	0.248	35544 (31368.0, 53409.0)	0.594	
Endpoint (E)	49608 (34422.0, 129492.0)		40314 (36066, 64800)		
Change (E-B)	13626 (-11634.0, 36402.0)		8508 (-20322, 25464)		0.606
**ΔAUC**_**cp**_ **(ng/mL)**					
Baseline (B)	132 (103.3, 195.0)	0.010	163 (95.25, 254.75)	0.026	
Endpoint (E)	269 (172.0, 319.0)		300 (277.0, 346.0)		
Change (E-B)	97 (57.0, 142.0)		145 (34.0, 212.0)		0.898
**ΔAUC**_**insulin**_ **/ ΔAUC**_**glucose**_					
Baseline (B)	1.2 (1.07, 2.14)	0.062	1.4 (0.92, 1.98)	0.003	
Endpoint (E)	1.9 (1.48, 4.03)		2.2 (1.40, 4.30)		
Change (E-B)	0.4 (-0.11, 2.89)		0.7 (0.42, 2.13)		0.450
**ΔAUC**_**cp**_ **/ ΔAUC**_**glucose**_					
Baseline (B)	0.03 (0.011, 0.041)	0.026	0.03 (0.015, 0.097)	0.013	
Endpoint (E)	0.05 (0.027, 0.099)		0.11 (0.054, 0.201)		
Change (E-B)	0.02 (0.008, 0.066)		0.07 (0.017, 0.181)		0.250
**IGI**_**60**_ **(pmol/ mmol)**					
Baseline (B)	126.8 (91.37, 212.46)	0.328	135.0 (60.34, 203.19)	0.059	
Endpoint (E)	204.0 (63.21, 407.27)		256.8 (119.26, 347.52)		
Change (E-B)	31.8 (-30.05, 297.33)		82.66 (-2.00, 203.87)		0.863
BIGTT-AIR (min*pmol/L)					
Baseline (B)	2372.9 (1761.27, 3438.48)	0.062	3010.1 (1871.95, 3722.19)	0.424	
Endpoint (E)	3864.7 (2844.36, 5035.23)		2527.1 (1863.77, 3453.72)		
Change (E-B)	1241.2 (-299.48, 2260.43)		-144.8 (-1893.53, 916.62)		0.133
**Insulin sensitivity**					
**QUICKI**					
Baseline (B)	0.32 (0.310, 0.347)	0.248	0.33 (0.311, 0.344)	0.110	
Endpoint (E)	0.31 (0.303, 0.328)		0.33 (0.315, 0.354)		
Change (E-B)	-0.01 (-0.029, 0.008)		0.01 (-0.005, 0.021)		0.047
**OGIS (ml/ min/ m** ^**2**^ **)**					
Baseline (B)	435 (356.5, 455.5)	0.790	438 (397.0, 463.0)	0.689	
Endpoint (E)	396 (315.0, 469.0)		446 (358.0, 488.0)		
Change (E-B)	-10 (-47.0, 55.0)		-1 (-82.0, 42.0)		1.00
**BIGTT-S (10** ^-5^*(min*pmol/L) ^**-1**^ **)**					
Baseline (B)	4.5 (2.52, 8.19)	0.374	6.4 (3.45, 9.23)	0.859	
Endpoint (E)	4.9 (1.31, 5.57)		5.3 (3.64, 9.13)		
Change (E-B)	-0.6 (-1.15, 0.60)		-0.3 (-1.29, 1.04)		0.699
Disposition index (OGIS* ratio of total AUC_**insulin**_ **over AUC**_**glucose**_ **)**					
Baseline (B)	25.7 x 10^3^ (22.13 x 10^3^, 32.45 x 10^3^)	0.021	24.1 x 10^3^ (19.64 x 10^3^, 33.78 x 10^3^)	0.477	
Endpoint (E)	33.7 x 10^3^ (32.51 x 10^3^, 44.60 x 10^3^)		29.6 x 10^3^ (21.07 x 10^3^, 42.44 x 10^3^)		
Change (E-B)	7.7 x 10^3^ (2.94 x 10^3^, 17.52 x 10^3^)		4.5 x 10^3^ (-3.88 x 10^3^, 10.70 x 10^3^)		0.171

Three women in the vitamin D group had IGT at baseline. At the end of the 6 months study, 1 reverted to normal while 2 others remained at the IGT level. In the placebo group, 1 woman with IGT normalized, 1 with IFG progressed to diabetes and 2 women who started with normal glucose response progressed to IGT.

### Effect of vitamin D supplementation pancreatic beta-cell function and insulin sensitivity

The basal pancreatic beta-cell function, as measured by fasting insulin increased by 20% in the vitamin D group and decreased by 18% in the placebo group (p = 0.034). Fasting C-peptide increased significantly in both arms (77.8% in vitamin D and 51% in placebo). This increased basal insulin secretion in the absence of changes in basal glucose implied improved basal pancreatic beta-cell function following supplementation of vitamin D. The total insulin secretion over 2 hours following the glucose challenge, as measured by AUC_insulin_ (total pancreatic insulin secretion) and AUC_cp_ (total insulin reaching the peripheral circulation) did not differ between groups.

The dynamic pancreatic response following glucose challenge did not show consistent change with vitamin D supplementation (Table 2). The BIGTT-AIR increased significantly by 52% in the vitamin D group. The IGI_60_ and global insulinogenic index increased but was not affected by vitamin D supplementation.

Vitamin D had no obvious effect on insulin sensitivity. No within-group changes were observed for QUICKI, OGIS and BIGTT-S. A borderline between-groups difference (p = 0.047) was noted for QUICKI but not for OGIS and BIGTT-S. The disposition index increased significantly in the vitamin D group (Table 2).

### Effect of vitamin D supplementation on markers of cardio-metabolic risk

Overall, vitamin D supplementation did not alter the markers of cardio-metabolic risk (Table 3). There were no significant between-group differences in changes in blood pressure, BMI, WHR, hsCRP, total cholesterol, LDL-cholesterol and HDL-cholesterol. The placebo group experienced a within-group decrease in BMI, systolic and diastolic blood pressures and LDL-cholesterol, while the vitamin D group saw a rise in triglyceride and triglyceride to HDL-cholesterol ratio after 6 months. These within-group differences may be due to chance findings given the small sample size.

**Table 3:** Changes in markers of cardio-metabolic risk among women with former GDM in both interventional and placebo groups before and after intervention.

	Vitamin D group (n = 13)		Placebo group (n = 13)		
	Median (25^th^, 75^th^ percentiles)	p-value difference within group	Median (25^th^, 75^th^ percentiles)	p-value difference within group	p-value changes(E-B) between groups
**Systolic blood pressure (mmHg)**					
Baseline (B)	123.0 (108.00, 130.50)	0.083	115.0 (112.00, 126.00)	0.013	
Endpoint (E)	116.0 (105.33, 126.67)		106.0 (97.67, 113.67)		
Change (E-B)	-3.0 (-12.67, 1.33)		-11.0 (-17.33, -5.0)		0.217
**Diastolic blood pressure (mmHg)**					
Baseline (B)	81.0 (70.00, 83.50)	0.625	72.0 (68.50, 83.00)	0.023	
Endpoint (E)	76.8 (72.67, 84.00)		67.0 (64.67, 73.33)		
Change (E-B)	2.0 (-5.33, 3.67)		-6.0 (-12.67, 0.33)		0.065
**Body mass index (kg/ m** ^**2**^ **)**					
Baseline (B)	26.5 (21.95, 30.67)	0.929	28.8 (25.55, 30.96)	0.026	
Endpoint (E)	25.8 (23.61, 30.36)		27.8 (25.99, 29.99)		
Change (E-B)	0.1 (-0.77, 0.78)		-0.6 (-1.04, 0.14)		0.133
**Waist-to-hip circumference ratio**					
Baseline (B)	0.89 (0.808, 0.968)	1.0	0.89 (0.838, 0.925)	0.155	
Endpoint (E)	0.90 (0.835, 0.960)		0.90 (0.869, 0.965)		
Change (E-B)	0.02 (-0.054, 0.053)		0.04 (-0.014, 0.077)		0.151
**Highly sensitive C-reactive protein (μg/mL)**					
Baseline (B)	2.4 (1.45, 7.60)	0.563	2.2 (0.90, 7.50)	0.594	
Endpoint (E)	1.8 (0.50, 3.50)		3.7 (0.40, 4.90)		
Change (E-B)	-0.2 (-2.20, 1.00)		0 (-2.10, 1.40)		0.748
**Urinary microalbumin-creatinine ratio (mg/mmol)**					
Baseline (B)	1.0 (0.53, 2.70)	0.399	1.0 (0.70, 1.30)	0.722	
Endpoint (E)	0.9 (0.58, 1.33)		0.8 (0.60, 1.60)		
Change (E-B)	0 (-1.55, 0.20)		-0.1 (-0.70, 0.90)		0.766
**Serum total cholesterol (mmol/L)**					
Baseline (B)	5.6 (4.65, 6.15)	0.423	4.7 (4.20, 5.45)	0.181	
Endpoint (E)	5.6 (4.60, 5.80)		4.4 (4.10, 5.10)		
Change (E-B)	-0.2 (-0.90, 0.50)		-0.5 (-0.70, 0.30)		0.921
**Serum low-density lipoprotein (mmol/L)**					
Baseline (B)	3.9 (3.05, 4.20)	0.327	3.0 (2.60, 3.80)	0.016	
Endpoint (E)	3.6 (2.90, 3.90)		2.5 (2.30, 3.60)		
Change (E-B)	-0.4 (-0.70, 0.20)		-0.3 (-0.43, -0.05)		0.804
**Serum high-density lipoprotein (mmol/L)**					
Baseline (B)	1.2 (1.05, 1.50)	0.256	1.2 (1.00, 1.30)	0.831	
Endpoint (E)	1.3 (1.10, 1.40)		1.2 (1.00, 1.60)		
Change (E-B)	-0.1 (-0.20, 0)		-0.1 (-0.15, 0.15)		0.693
**Serum triglyceride (mmol/L)**					
Baseline (B)	1.1 (0.80, 1.30)	0.049	1.0 (0.85, 1.20)	0.383	
Endpoint (E)	1.2 (0.90, 1.60)		0.9 (0.60, 1.30)		
Change (E-B)	0.1 (0, 0.50)		-0.20 (-0.400, 0.020)		0.041
**Serum triglyceride to high-density lipoprotein ratio**					
Baseline (B)	0.90 (0.558, 1.039)	0.021	0.91 (0.615, 1.154)	0.285	
Endpoint (E)	1.14 (0.600, 1.375)		0.85 (0.500, 1.083)		
Change (E-B)	0.20 (0.033, 0.375)		-0.27 (-0.425, 0.149)		0.024

## Discussion

This study highlights a high prevalence of vitamin D insufficiency among young women of Malaysian origin with former GDM and their high risk of progression to diabetes. Supplementation with vitamin D at 4000 IU daily for six months resulted in improved 25(OH)D levels and improved basal pancreatic beta-cell function when compared with placebo. There appeared to be no similar effect on the dynamic pancreatic function. While the improved BIGTT-AIR suggests amelioration, this was not supported by the other indices of dynamic pancreatic beta-cell function including IGI_60_ and the global insulinogenic index.

In our study, vitamin D replacement did not result in significant change in insulin sensitivity. Previous randomized controlled studies showed that vitamin D, when given in similar dose and duration, improved hepatic insulin sensitivity in insulin-resistant South Asian women [13] and obese adolescents [23]. A single injection of 300,000 IU vitamin D3 at 3–10 days after delivery reduces indices of insulin resistance in mothers with recent pregnancy complicated by GDM [24]. On the other hand, a study in obese African Americans [25], supplementation of vitamin D worsened hepatic insulin sensitivity. In observational studies, higher serum 25(OH)D and lower HOMA%IR were observed in Caucasian Americans but not in African Americans [4, 26]. These seeming conflicting results suggest that the effect of vitamin D on insulin sensitivity may be different in different ethnic groups. In our study, vitamin D replacement did not result in significant change in markers of peripheral insulin sensitivity, including OGIS and BIGTT-S. While the between-group difference in QUICKI may imply that vitamin D supplementation decreased hepatic insulin sensitivity, the marginal p-value could be equally likely due to the scattering of data in a small sample size. Our study included Malaysian women of three different ethnic groups and our sample size was inadequate to differentiate effect among the different ethnic groups; however, the similar baseline characteristics seem to exclude an effect of ethnicity within Malaysian women.

Although OGIS and ratio of AUC_insulin_ over AUC_glucose_ did not improve significantly, their combination (the so-called disposition index) increased significantly in the vitamin D group, suggesting indeed an amelioration of the compensatory mechanism of insulin resistance by insulin release [22]. This is supported by an improvement in the glucose tolerance and a significant drop in HbA_1c_ of 2 mmol/mol (0.2%) in the vitamin D group, but not in the placebo arm.

The overall trend suggests that vitamin D may have beneficial effect on glucose metabolism in women with previous gestational diabetes and hypovitaminosis D, by predominantly improving the basal pancreatic beta-cell function. This observation will need to be verified in a larger trial of longer duration. Contrary to observational data, supplementation of vitamin D failed to result in significant changes in markers of cardio-metabolic risk of blood pressure, lipid profile, hsCRP and MCR in this interventional study.

The high prevalence of vitamin D insufficiency despite the tropical climate and lack of seasonal variation is alarming. Inadequate intake from diets low in vitamin D, practice of deliberate sun avoidance and foetal utilization through previous pregnancy and lactation are all possible contributing factors. In addition, darker skin pigmentation may be associated with decreased vitamin D conversion [27]. This is important in our globalized world, because people of tropical origin going–for instance–to Northern Europe will lack sun light even more, potentially worsening their hypovitaminosis. On the other hand, Powe et al recently demonstrated that racial difference in common genetic polymorphism between black and white Americans contributes to variation in the levels of vitamin D-binding protein and bioavailability of 25(OH)D [28]. The vitamin D-binding protein was not incorporated into the assessment of vitamin D status in our multi-ethnic study population.

The strength of this study lied in the randomized placebo-controlled prospective design, in a well-defined group of individuals at high risk of progression to diabetes, the use of high dose of vitamin D over substantial duration, conduct of 2-hour OGTT which enabled use of various validated modeling to estimate insulin sensitivity and pancreatic function and the adjustment for confounder of physical activity. However, it was limited by the small number of participants of different ethnicities studied. A larger study with longer duration, including sufficient number of women with former GDM of different racial groups will be needed to explore if different races have different optimal serum concentration of 25(OH)D to improve glucose homeostasis.

## Conclusions

This study has demonstrated that 6 months supplementation of vitamin D in Asian women with previous gestational diabetes and hypovitaminosis D resulted in improved basal pancreatic beta-cell function, unchanged dynamic pancreatic beta-cell function and overall amelioration of the metabolic state as shown by an improved DI and HbA_1c_. There was no effect on markers of cardio-metabolic risk. Further mechanistic studies exploring the role of vitamin D supplementation on glucose homeostasis among different ethnicities may be needed to better inform future recommendations for these women with former GDM at high risk of both hypovitaminosis D and future diabetes.

## Supporting Information

S1 CONSORT Checklist CONSORT Checklist. (DOC)

S1 Protocol Trial Protocol. (DOCX)

S1 Study Dataset Study data set and variable explanatory information. (RAR)
