# Correction: Impact of Vitamin D Replacement on Markers of Glucose Metabolism and Cardio-Metabolic Risk in Women with Former Gestational Diabetes—A Double-Blind, Randomized Controlled Trial

**DOI:** 10.1371/journal.pone.0136003

**Published:** 2015-08-14

**Authors:** Toh Peng Yeow, Shueh Lin Lim, Chee Peng Hor, Amir S. Khir, Wan Nazaimoon Wan Mohamud, Giovanni Pacini

In the seventh sentence of the Abstract, the “-”(negative) symbol is absent. The correct sentence is: Supplementation, when compared with placebo, resulted in increased vitamin D level (+51.1nmol/L vs -0.2nmol/L, p<0.001) and increased fasting insulin (+20% vs -18%, p = 0.034).

## References

[1] Yeow TP, Lim SL, Hor CP, Khir AS, Wan Mohamud WN, Pacini G (2015) Impact of Vitamin D Replacement on Markers of Glucose Metabolism and Cardio-Metabolic Risk in Women with Former Gestational Diabetes—A Double-Blind, Randomized Controlled Trial. PLoS ONE 10(6): e0129017 doi: 10.1371/journal.pone.0129017 26057782 10.1371/journal.pone.0129017 PMC4461258

