# Supplementary material for: Impact of Vitamin D Replacement on Markers of Glucose Metabolism and Cardio-Metabolic Risk in Women with Former Gestational Diabetes—A Double-Blind, Randomized Controlled Trial
Source: PLoS One. 2015 Jun 9;10(6):e0129017. doi: 10.1371/journal.pone.0129017 (PMC4461258; doi:10.1371/journal.pone.0129017)
Supplement: S1 Protocol — (DOCX) [file pone.0129017.s002.docx]

**PROTOCOL TITLE: The effects of vitamin D supplementation on blood glucose and markers of metabolic syndrome in women with Vitamin D deficiency and previous Gestation Diabetes Mellitus**

| **Document author(s)** | **Reviewed and approved by** |
| --- | --- |
| **Yeow Toh Peng**  Date: 13/10/2011 | Date**:** |

| **Rev #** | **Section** | **Revision Date** | **Reason for Revision** | **Mgmt Rep** |
| --- | --- | --- | --- | --- |
| 1 | All |  | First time issued |  |
| 2 | 1. Protocol 2. Sample size calculation 3. Safety evaluation 4. ICF | 30^th^ June 2010 | In response to comment from reviewer |  |
| **3** | **Study title**  **Sect 1: Synopsis**  **Sect 6: Treatment and study procedure** | **3^rd^ March 2011** | **-Revision of study initiation and end date ( change due to delay in obtaining study drug)**  **-Change of vitamin D supplier from Carlson to Blackmores** |  |
| **4** | 1. **Section 1: Inclusion and Exclusion criteria** 2. **Investigations** 3. **Timing for visits** | **13^th^ October 2011** | **-inclusion criteria widened to include women up to 48 months post GDM pregnancy**  **-Exclusion criteria of “breast feeding” defined**  **-HsCRP, urinary MCR and Lipid profile included as measurement of change in insulin resistance**  **Participants of this study are often mothers of young children and may have difficulty attending study visits too frequently at strict timing. In order to facilitate attendance, following changes are made:**  **-Visit 2 made into optional so that participants have the option of having results discussed over phone and thus reduce frequency of visit to CRC.**  **-Timing of visit 3 to 5 made more flexible by addition of 1 week either side of allocated time.** |  |

**Study Title : “The effects of vitamin D supplementation on blood glucose and markers of metabolic syndrome in women with Vitamin D deficiency and previous Gestation Diabetes Mellitus”**

**Study initiation date :** …1^st^ June 2011……………….

**Study completion date :** …30^th^ November 2011…………….

**Date** **of protocol :** …30/06/2010…………….

**Amendment number(s) and date :** ……3rd amendment, date 13^th^ October 2011

**Author(s)** : Yeow Toh Peng………………..

Author’s and Reviewer’s signature and date:

| **Protocol Author** | **Reviewed and approved by** |
| --- | --- |
| Signature:  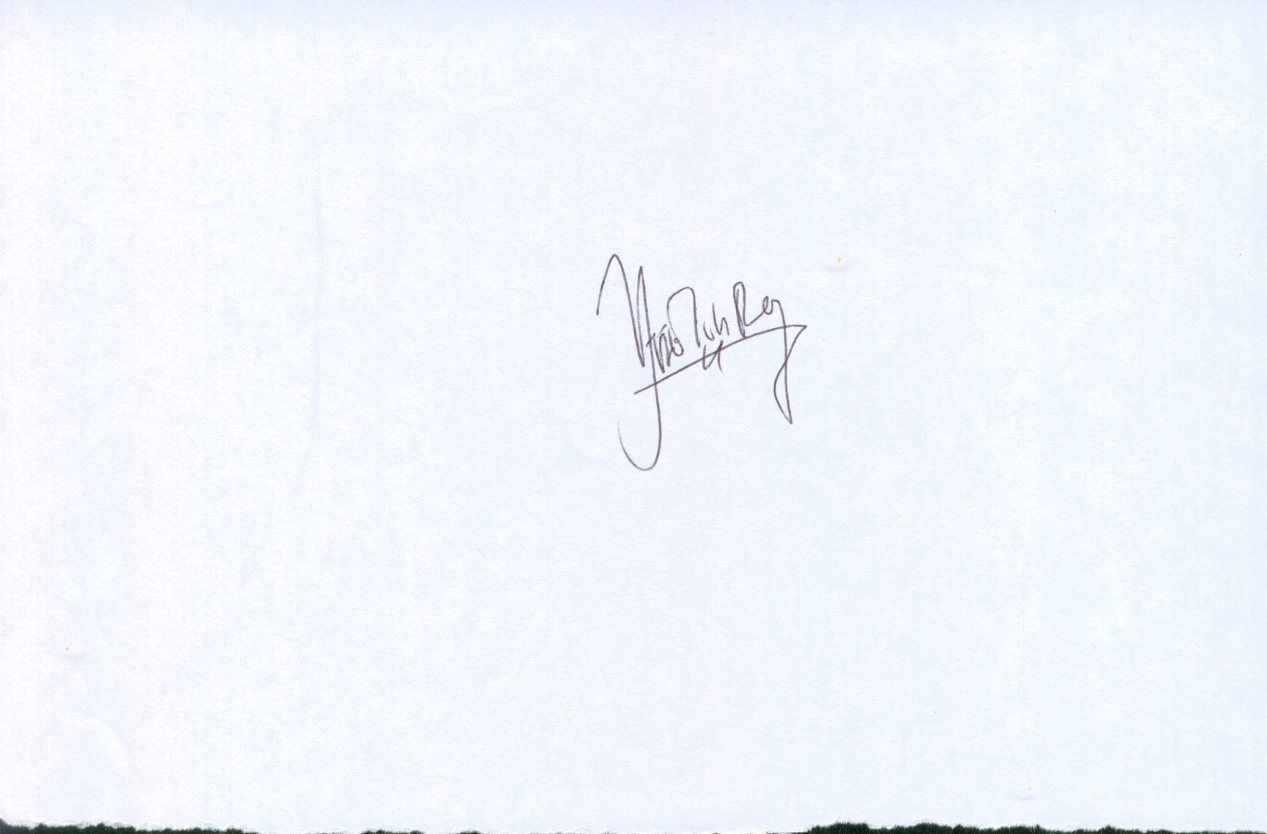 | Signature: |
| Date: 30/06/2010 | Date: |

This protocol incorporates the following amendment(s):

| **Amendment No.** | **Date of Amendment** | **Initials of Principal Investigator** |
| --- | --- | --- |
| 1 | 30^th^ June 2010 | TP |
| 2 | 3^rd^ March 2011 | TP |
| 3 | 13^th^ October 2011 | TP |

| **Confidentiality Statement**  **May not be used, divulged, published or otherwise disclosed without the written consent of …………………………….** |
| --- |

# SYNOPSIS

| **Title of study:**  “The effects of vitamin D supplementation on glucose metabolism and markers of metabolic syndrome in women with Vitamin D deficiency and previous Gestation Diabetes Mellitus” |
| --- |
| **Sponsor:** |
| **Clinical Phase:** |
| **Investigators:**  Investigators will be endocrinologists practicing in Penang General Hospital |
| **Study period:** 1 years  Planned date of first subject enrolment: 1^st^ June 2011……………..  Planned date of last subject completed: 30^th^ November 2011………………. |
| **Objectives:**   - Primary objective:   - To establish efficacy of Vitamin D supplementation compared to Placebo with respect to Glucose Metabolism in female subjects with Vitamin D deficiency and previous Gestational Diabetes - Secondary objectives :   - To establish effect of Vitamin D supplementation with effect to markers of metabolic syndrome namely- BMI, WHR and BP   - To ascertain if 4000iu of Vitamin D3 replacement is adequate in raising plasma 25 (OH) Vit D level to > 80 nmol/L |
| **Methodology:**  This is a randomised, double blind, placebo controlled, parallel group design |
| **Number of patients:**  It is planned to randomise an estimated total of 26 adult patients into this study, with 13 patients randomised into each treatment group. |
| **Number of centres:**  1 |
| **Inclusion criteria:**   - Women with Gestational Diabetes (GDM) on the most recent pregnancy– between 6 and 48 months post partum at time of recruitment to study - Previous GDM defined as-   - Fasting plasma glucose ≥ 7.0 mmol/l or 2-h plasma glucose ≥ 7.8 mmol/l on 75g OGTT performed during gestation OR   - As determined by treating physician during index pregnancy - With no plan to conceive again within the 6 months of study duration - With 75g OGTT at study baseline confirming either Normal respond, Impaired Fasting Glucose (IFG) or Impaired Glucose Tolerance (IGT) - Vitamin D deficiency at baseline – defined as Plasma 25-(OH) D concentration of between 15 and 50nmol/L |
| **Exclusion criteria:**   - Pregnancy - Breast-feeding (defined as women who are providing full breast feeding) - Known Type 1 or Type 2 Diabetes - Newly diagnosed diabetes as shown by study baseline 75g OGTT (WHO definition) - Use of any oral hypoglycaemic agent or Insulin - Concomitant use of antituberculosis medications, antiepileptic medication, vitamin D or calcium supplement. - Intolerant or allergy to Vitamin D supplementation - Women with renal disease or serious co-morbid illness - Severe vitamin deficiency at baseline- as defined as plasma 25(OH) D concentration of < 15 nmol/L |
| **Test treatment, dose and mode of administration:**  Treatment: Vitamin D 3 capsule  Dose: 4000iu  Route: oral once a day |
| **Duration of treatment with study medication:**  6 months |
| **Criteria for evaluation:**   1. Efficacy parameter(s)  - Primary criterion   - Change in glucose metabolism as assessed by area under curve for glucose (AUCgluc) and insulin (AUC ins) post 75 g OGTT   - Change in Insulin sensitivity index as calculated by HOMA –IR^28^ - Secondary criteria   - Change in Insulin Sensitivity index as calculated by QUIKI^27^   - Change in dynamic Insulin Sensitivity using OGTT Insulin Sensitivity (OGIS)^25,26^   - Change in Beta cell function as evaluated by ratio of AUCins to AUCgluc^29^   - Change in Blood pressure (BP), lipid profile, HsCRP, Urinary microalbumin-creatinine ratio, Body Mass Index (BMI) and Waist Hip Ratio (WHR)   - Change in plasma 25-OH- Vitamin D level pre and post replacement |
| **Statistical methods:**   1. Sample size and power considerations   By using nQuery Advisor version 6.01, from paper written by Von Hurst et al (Vitamin D supplementation reduces insulin resistance in South Asian women living in New Zealand who are insulin resistant and vitamin D deficient - a randomised, placebo-controlled trial )^31^, a sample size of 9 will have 95% power to detect a difference in means of 0.700 (e.g. the differences between Group 1 mean, µ1, of 0.800 and a Group 2 mean, µ2, of 0.100), assuming that the common standard deviation is 0.384, with a 0.050 two-sided significance level. By adding 40% dropped out patient in case of lost to follow up or unplanned pregnnacy, the total sample for 2 arms (placebo and treatment group) treatments is 26 patients (13 patients for each arms).  Green et al (Vitamin D status and its association with parathyroid hormone concentrations in women of child-bearing age living in Jakarta and Kuala Lumpur)^15^ found 60% of population to have 25 (OH) Vit D of < 50nmol/L. Assuming a similar prevalence exist in our population, we thus expect to screen at least 44 women to recruit 26 subjects for the study.  Green et al (Vitamin D status and its association with parathyroid hormone concentrations in women of child-bearing age living in Jakarta and Kuala Lumpur)^15^ found 60% of population to have 25 (OH) Vit D of < 50nmol/L. Assuming a similar prevalence exist in our population, we thus expect to screen at least 44 women to retain 26 subjects for the study. |

# LIST OF ABBREVIATIONS

AE Adverse event

BP Blood pressure

BMI Body Mass Index

CRC-Pg Clinical Research Centre, Penang Hospital

CRC-PMC Clinical Research Centre, Penang Medical College

CV Curriculum vitae

GDM Gestational Diabetes Mellitus

GCP Good clinical practice

HOMA-IR homeostasis model assessment-estimated insulin resistance

IEC Independent Ethics Committee

IFG Impaired Fasting Glucose

IGT Impaired Glucose Tolerance

ID Identification

IRB Independent Review Board

OGTT Oral Glucose Tolerance Test

OGIS Oral Glucose Insulin Sensitivity

QUIKI Quantitative insulin sensitivity check index

T2DM Type 2 Diabetes Mellitus

WHR Waist Hip Ratio

mmHg Millimetre mercury

SAE Serious adverse event

#

# INTRODUCTION AND BACKGROUND

**Introduction:**

Gestational diabetes Mellitus (GDM) is a transient state of carbohydrate intolerance occurring during pregnancy with full resolution post-partum. Diagnositc criteria is yet to be uniform but Malaysia has subscribed to the WHO criteria which is fasting plasma glucose of ≥ 7.0 mmol/L and/or 2 hour plasma glucose of ≥ 7.8 mmol/L following 75g OGTT.

GDM is related to both resistance to peripheral action of insulin and impairment of beta cell function made more prominent with hormonal change during pregnancy. Its presence alerts to heightened risk of development of Diabetes in future. 10-50% of women with GDM develop Diabetes Mellitus later on in life ^1^. Data from Malaysia suggest conversion rate as high as 7-9.9% per year^2^. Identification of effective prevention measure in this high risk group is of significant public health concern.

Insufficient level of Vitamin D is increasingly recognized to impact on a plethora of health condition ranging from bone health, to cancer, autoimmune disease, cardiovascular disease and diabetes. Vitamin D deficiency has been shown to cause insulin resistance and impaired pancreatic function.^3,4,5^ Vitamin D deficiency has also been shown to be a risk factor for future development of diabetes^6.7.8,9^ and data from cohort study demonstrate that higher intake of calcium and vitamin D is associated with reduced risk of development of T2DM^10^.

Vitamin D deficiency is more prevalent in women with gestational diabetes with low level correlating with insulin resistance^11,12,13^. Data from Australia suggest that 48% of women with gestational diabetes has vitamin D deficiency as defined by plasma 25 (OH) D level of < 50nmol/L^11^.

There is as yet no data on vitamin D status among women with GDM in Malaysia but there are reasons to suspect deficiency is prevalent. Study suggests that 60% of Malaysian women of child bearing age has vitamin D deficiency^15^. Inadequate intake from diet low in vitamin D, practice of deliberate sun avoidance and fetal utilization through previous pregnancy and lactation are all possible contributing factors. Darker skin pigmentation may be associated with decrease vitamin D conversion^14^.

There are circumstantial evidences to suggest Vitamin D replacement may be an effective preventive measure in this high risk group. Von Hurst et al showed that supplementation with 4000 iu Vit D3/day in population of south asian women with proven vitamin D deficiency was safe and improves insulin resistance^31^ . Other studies have yielded mix results—partly due to variable dose of supplementation and duration of follow up^16,17,18,19,20,21,22^. Some of the studies are of very short duration of less than 7 days^19,20,21^. Previous studies suggest a possible optimal window for vitamin D replacement to effectively influence glucose metabolism. Vitamin D supplementation was shown to have positive effect on glucose metabolism when introduced during early stage of glucose intolerance but no effect when used in patients with established chronic diabetes^21^. On the other spectrum, vitamin D supplementation was shown to improve glucose metabolism in subjects with impaired fasting glucose but not in normal volunteers^17.^  Lack of adequate dosing may have also accounted for failure of many previous studies to demonstrate beneficial effect of Vitamin D replacement. Scragg et al suggest that vitamin D supplementation need to raise blood 25(OH)D levels above 80 nmol/l because diabetes risk is lowest at this levelVitamin D^23^. .

.

Very little is known about vitamin D status of young GDM population in Malaysia. There has been as yet no study evaluating the role of vitamin D supplementation in reducing the future development of diabetes in this high-risk group of past GDM.

We have therefore design a study consisting of 2 phases. The first phase is to evaluate the prevalence of vitamin D deficiency in women with past GDM. The second phase involves recruitment of selected women into the vitamin D intervention trial.

**Hypotheses**

- There is a high prevalence of hypovitaminosis D in past GDM population
- Supplementation of Vitamin D in women with previous GDM and vitamin D deficiency will result in an improvement in glucose metabolism

**Rationale for study design**:

Overall rationale: Considering the high conversion rate from GDM to Type 2 diabetes, it is crucial to identify effective measure of diabetes prevention in this population. Vitamin D deficiency may be potentially an important contributing factor. The study will identify vitamin D deficiency in a group of women with previous GDM to examine if replacement can improve parameters of glucose metabolism. If we can demonstrate the protective effect of vitamin D replacement, it can be used as a simple and cheap solution to prevent future IGT or DM in this at risk group.

Rationale for inclusion exclusion criteria:

- Women who are 6 to 24 months post partum are chosen as immediate postpartum period may be associated with fluctuation in weight and therefore glucose metabolism. A high percentage of women may have already developed diabetes if identified too many years after GDM pregnancy.
- We exclude pregnant women. Vitamin D at dose of 4000iu/ day is safe in for maintainence or prevention of Vit D deficiency is safe and recommended in pregnancy ^24^. However, as the study involves use of placebo, subjects who are found to be pregnant during screening will be excluded from further participation of trial.
- We exclude women who are breastfeeding as they may require a higher dose of vitamin D to achieve adequate plasma vitamin D level, participation in blinded study involving use of placebo cannot be justified.
- There is no current consensus on optimal level of plasma 25(OH) D, though level below 50nmol/L is generally considered deficient by most authorities and linked to metabolic disturbance. There is no current consensus to the level of vitamin D deficiency below which is deemed unsafe. Many women with vitamin D deficiency is asymptomatic and never screened. As the study involves use of placebo, we have chosen arbitrarily to exclude women with plasma 25(OH)D level less than 15nmol/L. Women with symptomatic hypocalcaemia will be excluded and referred for treatment rather than recruited to receive potential placebo.
- Women with chronic renal or liver failure are excluded due to potential impairment in vitamin D activation and utilization.

Rationale for chosen treament regimen:

- The intervention consist of 25(OH)D3 supplement 4000 iu per day , or placebo, in the form of 2 oral capsules , for 6 months. Oral vitamin D is cheap and easy to take. Vitamin D3 is chosen over D2 as it is 3 times more potent and only a third of dose is required. Inadequate dosing is one of the reasons previous interventional studies has shown mixed result. Vitamin D dose given in prevention trials needs to be high enough to raise blood 25(OH)D levels above 80 nmol/l because diabetes risk is lowest at this level of Vitamin D^23^. Supplementation with 4000 iu Vit D3/day in population with proven vitamin D deficiency was safe and effective in raising the plasma 25(OH) D to desired level^31,32,33^.

Rationale for duration of study end point evaluation:

- An end point data of diabetes development will be more clinically relevant but will require a long study follow up duration and with it many other confounding factors of weight change, diet and exercise. We have therefore opted for a 6 months study where the confounding factors can be identified and accounted for.
- 75g OGTT with Glucose and Insulin measurement, with validated calculation of QUICKI, HOMA-IR and OGIS will be used as surrogate markers of impact of carbohydrate tolerance, Insulin resistance and Insulin secretion.

Potential Risks

- We will be using vitamin D3 replacement dose of 4000 iu/day and do not expect any side effects from this dose. Dose of 4000iu/day has been used without side effects in previous studies^31-33^.
- Synthesis of 1,25(OH)_2_D (the most active metabolite of vitamin D) is tightly regulated, vitamin D toxicity usually occurs only if excessive doses (prescription or megavitamin) are taken. Vitamin D 40,000 IU/day produces toxicity within 1 to 4 month in infants. In adults, taking 60,000 IU/day for several months can produce toxicity. In event of toxicity, marked hypercalcemia commonly causes symptoms. Treatment consists of stopping vitamin D, restricting dietary Ca, restoring intravascular volume deficits, and, if toxicity is severe, giving corticosteroids or bisphosphonates.

Reference: Martindale: The Complete Drug Reference

## Potential Benefits

Vitamin D deficiency is not routinely tested for due to its high cost. Our study can identify women with vitamin D deficiency and offer supplementation. Vitamin D replacement is of benefit as deficiency causes adverse effect on bone health. Studies have also suggested its role on many chronic illnesses for example common cancers, autoimmune diseases, infectious diseases and cardiovascular health^24^.

Patient are offered OGTT as part of the study, this may identify early clinically silent glucose intolerance or diabetes. Patients who are found to have already developed diabetes will be excluded from the phase 2 vitamin D intervention studies and referred for appropriate management.

# OBJECTIVES

## Primary objective:

To establish efficacy of Vitamin D supplementation compared to Placebo with respect to glucose metabolism in female subjects with Vitamin D deficiency and previous Gestational Diabetes

## Secondary objective(s):

To establish effect of Vitamin D supplementation on markers of metabolic syndrome namely BMI, WHR and BP

## Ancillary objective(s):

To establish effect of Vitamin D supplementation at 4000iu per day on raising plasma 25(OH) vit D level

# STUDY DESIGN

## Overall study design

The study consists of 2 phases: the first phase includes initial recruitment and screening. The second phase includes recruitment of selected individuals into the vitamin intervention trial.

The intervention trial is a prospective, randomized, double-blind, placebo controlled, parallel group trial to assess the efficacy of Vitamin D supplementation on blood glucose and markers of metabolic syndrome in women with Vitamin D insufficiency and previous Gestation Diabetes Mellitus. Study recruitment will cease when the pre determined number of subjects have been enrolled. Recruitment period for study is estimated to be 6 months

At least 44 subjects will be screened in order to recruit 26 subjects for the study.

**Visit 1: Screening:** Otherwise healthy women with previous GDM now 6 to 24 months post partum, who are not breastfeeding and has no plan to conceive again will be invited to participate in the trial.

All women will have screening assessment, which includes:

- Obtain consent
- Medical history – assessment of inclusion/ exclusion criteria
- Record of concomitant medications- intake of vitamin D will be prohibited within 3 months of screening
- Clinical examination- including
  - Blood pressure measurement- average of 3 readings (sitting)
  - BMI
  - Waist: Hip ratio
- Blood investigations: UE/creatinine, LFT, Calcium, lipid profile, HsCRP, Urinary microalbumin-creatinine ratio,—women with chronic renal or liver disease will be excluded
- 75g Oral Glucose tolerance test—with measurement for
  - Glucose and in at time 0,60,90 and 120 minutes—subject with OGTT showing fasting glucose > 7mmol/L and/or 2 hour glucose >11.1 mmol/L will be excluded from study
  - Insulin at time 0, 60, 90 and 120 minutes – only subjects with glucose and vitamin D level fulfilling inclusion criteria will have insulin sample send for measurement
- Blood tests for plasma 25(OH) D-
  - Subjects with level > 50nmol/L will be reassured and excluded from study
  - Subjects with level < 15nmol/L will be diagnosed with Vitamin D deficiency and excluded from study

**Visit 2: Discussion of Screening results (Optional visit)**

Discussion of results: Participants may have their results discussed over the phone or may choose to return to CRC to have their results discussed in person.

Subjects with normal glucose tolerance, IFT or IGT AND plasma vitamin 25(OH) D level of between 20 and 50 nmol/L (inclusive) will proceed with randomisation to either receive Vitamin D or Placebo

- Urine pregnancy test will be performed in women who fulfil criteria to proceed with phase 2 of study- pregnant women will be excluded from phase 2 participation

.

Subjects with normal glucose tolerance and with level of Vitamin D > 50nmol/L will be reassured and excluded from further participation of trial

Subjects with abnormal glucose tolerance (IFT or IGT) and Vitamin D level of > 50nmol/L will be excluded from further participation of trial and will be referred for appropriate follow up.

Subjects with fasting glucose of > 7mmol/L and/or 2 hour glucose >11.1 mmol/L will be referred back to primary health care for repeat assessment to confirm or rule out diagnosis of Diabetes.

Subjects with vitamin D level of <15nmol/L will be referred back to primary health care for appropriate assessment.

Subjects found to have abnormal renal, liver and bone function test will be referred back to primary health care for appropriate assessment.

Eligible subjects will be invited to proceed with study by returning for Visit 3.

**Visit 3: Randomisation:**

Randomization assessment will include:

- Record of urine pregnancy test results
- Record of concomitant medications- ensure patient has not been started on vitamin D in the interim between screening and randomization assessment
- Physical activity are quantified using the validated Paffenbarger Physical Activity Questionnaire^30^
- Blood pressure measurement- average of 3 readings (sitting)

Subjects eligible to proceed with study will be randomised to either receive Vit D3 4000iu per day or placebo.

**Visit 4,5, :Follow up visits:**

All subjects will be seen every 8 weeks (+/-1 week) to be dispensed supply of Vitamin D or placebo. Compliance will be checked with pill count. Any side effects will be monitored.

At Visit 4, blood sample will be taken for calcium, phosphate and albumin

**Visit 6: Assessment at study completion:**

At the end of 6 months- all subjects will attend fasting and have

- Repeat clinical examination- including
  - Blood pressure measurement- average of 3 readings (sitting)
  - BMI
  - Waist: Hip ratio
- Repeat 75g Oral Glucose tolerance test—with measurement for
  - glucose and Insulin at time 0,60,90 and 120 minutes
- Repeat Blood tests for plasma 25(OH) D, calcium, phosphate and albumin, lipid profile, HsCRP and Urinary microalbumin-creatinine ratio.

The study will be conducted in Clinical Research Unit of Hospital Pulau Pinang.

## Schematic diagram of study design:

Design of a randomized, controlled, double-blind clinical trial

Screening Results Randomisation Trial Observation Study ends

Assessment Treatment Period

|  |  |  | Vit D3 4000 iu PO od |  |  |  |  |
| --- | --- | --- | --- | --- | --- | --- | --- |
|  |  |  | Placebo |  |  |  |  |

| Visit No. 1 | 2(optiona) | 3 | 4 | 5 |  | 6 |
| --- | --- | --- | --- | --- | --- | --- |
| Timeline -2wk | -1wk | 0 (+/-1) wk | 8(+/-1)wk | 16(+/-1)wk |  |  |

## Study population

### *Inclusion criteria*

a) Provision of written consent by subject

b) Female patients who fullfill ALL of criteria below

- - Previous Gestational Diabetes-according to WHO criteria or as documented on medical record
  - 6-48 months postpartum from GDM pregnancy
  - Negative Pregnancy test at screening
  - With no plan to conceive again within the 6 months of study duration
  - With 75g OGTT at baseline confirming either normal respond, IFT or IGT
  - Vitamin D deficiency at baseline – defined as Plasma 25-(OH) D concentration of between 15 and 50nmol/L inclusive

### *Exclusion criteria*

1. Inability or unwillingness to comply with the requirements of the study protocol
2. inability or unwillingness to provide written consent
3. Use of Vitamin D supplementation within 3 months of study of screening
4. History of active drug/alcohol dependence or abuse.

For phase 2 of study (intervention)

1. 75g OGTT at baseline confirming established diabetes
2. Vitamin D at screening of > 50 nmol/L or < 15nmol/L
3. History of intolerant or allergy to Vitamin D supplementation
4. Pregnancy
5. Breastfeeding (defined as full breast feeding)
6. Chronic Renal or liver failure, hypercalcaemia, hypocalcaemia or other medical condition (eg. concomitant use of antituberculosis treatment, antiepileptic medications or calcium supplementation) that, in the investigator’s judgement, may be associated with increased risk to the subject or may interfere with study assessments or outcomes.

### *Recruitment and Screening*

Potential participants will be identified and recruited for the study from the following sources:

- Investigator
- Co-investigators
- Physician referrals
- Institutional database

Screening – birth record from Penang General hospital Maternity Unit will be used to identify women with GDM who has delivered 6-24 months prior to study commencement. Suitable women are invited for screening

### *Subject withdrawal & drop-out*

Subjects are free to withdraw from the study at any time for any reason. Subjects may also be withdrawn from the study at any time at the discretion of the investigator.

### *Procedures for handling withdrawal*

Subjects who withdraw or are withdrawn from the study should:

- Have the reason(s) for their withdrawal recorded
- Be asked about the presence of any AEs
- Be seen by an investigator and all final assessments will be performed and recorded
- Have study treatment returned.

# TREATMENT AND STUDY PROCEDURES

## Description of study drug/intervention and comparator

Vitamin D3 capsule in the form of Blackmore’s Vitamin D3 1000iu soft gel capsule will be used. Each softgel capsule will contain 1000iu of Vitamin D3. Patient enrolled in study will take daily- 4 softgel capsules of vitamin D3 or matching placebo.

### *Storage*

Vitamin D3 / Placebo can be kept at room temperature and only accessible to authorised study personnel.

### *Dispensing*

Each patient will be dispensed sufficient medication for 8 weeks of therapy.

Upon dispensation, the investigator will record the following:

- subject ID and initials
- date dispensed,
- total bottles dispensed, batch number and expiry date of product

### *Accountability*

The investigator or designee must maintain current and accurate record of the receipt, inventory and dispensing, including shipping invoices, of all study supplies.

Accountability logs must be available for inspection at any time.

## Concomitant medication/treatment

- Vitamin D intake are not allowed within 3 months of screening period
- Use of any oral hypoglycaemic agents not allowed
- All other concomitant medicines/therapies are recorded and permitted during the study

## Blinding & Unblinding procedures

This is a double blind study- All subjects and assessors will be blinded to the study medication during the trial. Independent central block randomization will be done with help of CRC Hospital Pulau Pinang In the event that an AE or pregnancy occurs for which knowledge of the identity of the test drug is necessary to manage the subject's condition, blinding may be broken and patient identified to be on either Vitamin D or Placebo.

## Assessment of compliance

Accountability and subject compliance will be assessed by maintaining adequate "drug dispensing" and return forms. Subjects will be required to return all unused study medication, and the pill count will be entered into the CRF.

# EFFICACY & SAFETY ASSESSMENTS

## Assessment of efficacy

Efficacy of Vitamin D replacement will be assessed with parameters below:

- Primary criterion
  - Change in glucose metabolism as assessed by area under curve for glucose (AUCgluc) and insulin (AUC ins) post 75 g OGTT
  - Change in Insulin sensitivity index as calculated by HOMA –IR^28^
- Secondary criteria
  - Change in Insulin Sensitivity index as calculated by QUIKI^27^
  - Change in dynamic Insulin Sensitivity using OGTT Insulin Sensitivity (OGIS)^25,26^
  - Change in Beta cell function as evaluated by ratio of AUCins to AUCgluc^29^
  - Change in Blood pressure (BP), Body Mass Index (BMI) and Waist Hip Ratio (WHR)
  - Change in fasting lipid profile, HsCRP and urinary Microalbumin creatinine ration
  - Change in plasma 25-OH- Vitamin D level pre and post replacement

## Assessment of safety

Safety, tolerability and compliance will be assessed with 6 weekly assessments which consist of:

- 8 weekly dispensing of Vitamin D/ Placebo
- Pill count as measure of compliance
- Monitoring and recording of all adverse events
- Serum calcium and phosphate will be checked after 8 weeks of supplementation

### *Adverse Events (AE)*

Vitamin D dietary supplementation may be detrimental in persons already receiving an adequate intake through diet and exposure to sunlight. Minimum of 2000iu of Vit D3 is the recommended replacement dose for patients with vitamin D deficiency. No adverse events are anticipated for this study using 4000iu Vit D3 or placebo in women with documented vitamin D insufficiency.

Excessive intake of vitamin D (50,000 iu daily for a few months) can lead to development of hyperphosphataemia or hypercalcaemia. Associated effects include hypercalciuria, ectopic calcification, renal and cardiovascular damage. Symptoms of overdosage include anorexia, lassitude, nausea and vomiting, constipation or diarrhoea, polyuria, nocturia, sweating, headache, thirst, somnolence and vertigo. Hypersensitivity reactions can occur.

(Source: Martindale : The complete drug reference)

The 8 weekly dispensing of study meds will allow for monitoring and reporting of any unforeseen adverse event. Subjects are instructed to contact via phone for earlier review in event of AE or intolerance to study meds. Patient with intolerance of vitamin D leading to poor compliance will be excluded from trial.

### *Treatment and follow up of AE*

Treatment of any AE is at the sole discretion of the investigator who should follow up subjects with AE until the event has resolved, until the condition has stabilised, the subject is lost to follow-up, or the adverse events are otherwise explained. Appropriate medical care should be arranged for the patient. Abnormal tests should be repeated until they return to baseline levels or an adequate explanation of the abnormality has been found. Any follow up information should be reported as soon as it becomes available.

- - - - - Treatment of over-dosage

Significant overdose of Vitamin D occurs with very high intake. In adults, toxicity occurs with more than 50,000 IU/day of Vitamin D for several months.

Suspicion of overdose will be referred to study doctor for further assessment.

Unblinding will be done at discretion of study doctor to allow further management.

- - - - - Pregnancy

Vitamin D at dose of 4000iu/ day is safe in for maintainence or prevention of Vit D deficiency and recommended in pregnancy ^24^.

However, as the study involves use of placebo, subjects who are found to be pregnant during screening will be excluded from further participation of trial. Hormonal changes during pregnancy may also alter glucose metabolism and potentially interfere with accuracy of results.

Only patient with no active plan to get pregnant again within 6 months of study participation will be recruited. In the event of unplanned pregnancy after randomization, treatment of subject will be unblinded. Pregnant subjects will be excluded from further participation in trial and referred for appropriate follow up according to local practice.

# STUDY CONDUCT

## Study visits and procedures

Any deviation from the study procedures described below will be noted in study document

### *Screening visit*

Otherwise healthy women with previous GDM now 6 to 24 months post partum, who are not breastfeeding and has no plan to conceive again within 6 months from recruitment will be invited to participate in the trial.

All women will have screening assessment which includes:

- Obtain consent
- Subject's demography: date of birth/age, sex, race, height (cm) and weight
- Background and medical history
- Record of concomitant medications- patient taking medications that may interfere with outcome of vitamin D will be excluded- eg antiepileptic, antituberculosis and calcium supplement. intake of vitamin D is prohibited within 3 months prior to screening
- Assessment of inclusion/ exclusion criteria
- Clinical examination- including
  - Complete physical examination
  - Body weight (kg) and height (cm), no shoes in light clothing
  - Blood pressure will be measured in the sitting position after 5 minutes rest. 3 measurements will be taken with a 2 minute interval between measurements- average of 3 measurements will be recorded
- Blood investigations: UE/Creatinine/LFT/ Calcium/ phosphate/ fasting lipid profile/HsCRP/Urinary Microalbumin creatinine ratio and plasma 25 (OH) D level
- 75g oral glucose tolerance test- with measurement for glucose and insulin at 0, 30,90 and 120 mins

### *Visit 2 for discussion of result (Optional CRC visit)*

Results from screening visits will be discussed- participant have the option of attending CRC to have results discussed in person and may choose to have results discussed over the phone.

Subjects not eligible for further participation in study will be referred for appropriate care

Subjects eligible to proceed to randomization will have urine pregnancy test done

### *Randomization visit*

The following data will be collected at randomization visit:

- Record of urine pregnancy test results
- Record of concomitant medications- ensure patient has not been started on vitamin D in the interim between screening and randomization assessment
- Physical activity are quantified using the validated Paffenbarger Physical Activity Questionnaire^30^

This is a double blinded study. Subjects eligible to proceed with study will be randomly allocated to either Vitamin D3 4000iu/ day Placebo in a 1:1 ratio. Randomization to either treatment arm will be done through central independent randomization with help of CRC hospital Pulau Pinang.

### *8.1.4 .Visit 4*

Visit 4 occur at 8 (+/-1) weeks post randomization -

The investigator will perform the following procedures:

- - Record any change of dose administration of study treatment
  - Record any AE or SAE
  - Dispense study treatment
  - Measure serum calcium, phosphate and albumin level

### *8.1.5.Visit 5*

Visit 5 occur at 16 (+/-1) weeks post randomization -

The investigator will perform the following procedures:

- - Record any change of dose administration of study treatment
  - Record any AE or SAE
  - Dispense study treatment

### *8.1.5.Visit 6*

This is the final visit- all subjects will attend fasting and have

- Repeat clinical examination- including
  - Blood pressure measurement- average of 3 readings (sitting)
  - BMI
  - Waist: Hip ratio
- Repeat 75g Oral Glucose tolerance test—with measurement for
  - glucose and Insulin at time 0,60,90 and 120 minutes
- Repeat Blood tests for plasma 25(OH) D, calcium, phosphate, albumin, Fasting lipid profile, HsCRP and Urinary microalbumin creatinine ratio.

Study visits schedule and procedures are summarised in the table below:

|  | **Screening** | **Assessment of results (optional CRC visit)** | **randomization** | **Treatment** | | **End** | |
| --- | --- | --- | --- | --- | --- | --- | --- |
| **Visit** | **1** | **2** | **3** | **4** | **5** |  | **6** |
| **Timeline** | **-2 wks** | **-1 wk** | **0 +/-1 wks** | **8 +/-1 wks** | **16 +/-1 wks** |  | **24**  **wks** |
| **Procedures** |  |  |  |  |  |  |  |
| Check eligibility | X | X | X |  |  |  |  |
|  |  |  |  |  |  |  |  |
| Informed consent | X |  |  |  |  |  |  |
| Randomisation |  |  | X |  |  |  |  |
| Patient demographics & medical history | X |  |  |  |  |  |  |
| Clinical examination- incl BP/ WHI/BMI  Paffenburger physical activity score | X |  | X |  |  |  | X |
| Lab test:  UE/ Creatinine/ LFT  Calcium, phosphate and albumin  Fasting lipid profile  HsCRP  Urinary Microalbumin Creatinine Ratio  2 hour 75g OGTT with glucose and insulin | X  X  X  X  X  X |  |  | X |  |  | X  X  X  X |
| Plasma 25(OH) D | X |  |  |  |  |  | X |
| Urine Pregnancy test |  | X |  |  |  |  |  |
|  |  |  |  |  |  |  |  |
| Report AE and SAE |  |  | X | X | X |  | X |
| Dispense study treatment |  |  | X | X | X |  | X |
| Complete relevant section of CRF | X |  | X | X | X |  | X |

## Criteria for stopping subject treatment

Subjects can withdraw consent from study at any time point without compromising care

Subjects who became pregnant during duration of study will be withdrawn and referred for follow up as per local practice

Subjects who are deemed inappropriate for further continuation at discretion of study co-ordinator will be withdrawn from study and reason documented

## Dropouts and withdrawals

50% drop out rate is included in statistical calculation

Reasons for drop outs will be documented as far as possible

## Sample handling and analysis

## Collection

105 ml of blood will be drawn for through the 6 months study duration- consisting of:

50 ml of blood will be drawn at screening visit (wk -2) for –

UE, creatinine, LF, Calcium and phosphate, fasting lipid profile, HsCRP (5ml)

plasma 25(OH) vit D ( 5ml)

OGTT at time 0, 60,90 and 120 min for glucose ( 20ml) and insulin ( 20ml)

5 ml of blood will be drawn at visit 4 (wk 8) for-

Calcium, phosphate and albumin (5 ml)

50ml of blood will be drawn at study completion ( wk 24) for-

Calcium, phosphate and albumin, fasting lipid profile, HsCRP (5ml)

plasma vit D ( 5ml),

OGTT at time 0, 60,90 and 120 min for glucose (20ml) and insulin ( 20ml)

### Shipment of samples

- Plasma UE, creatinine, LFT, Calcium, phosphate, Lipid profile and glucose will be send for immediate analysis in biochemistry laboratory in Penang General Hospital
- Plasma 25(OH)D level will be send for immediate analysis in biochemistry lab in Island hospital, Penang
- HsCRP and Insulin during 75g OGTT will be collected in EDTA bottle, spinned down and sample stored in -20°C fridge. Samples will be shipped to IMR lab for analysis when sufficient samples are accumulated

9 STUDY QUALITY ASSURANCE AND DATA MANAGEMENT

## 9.1 Study Quality Assurance

### *Pre-study*

- SOPs will be prepared for CRC PMC
- The investigators will review the protocol and study procedures with study stuff
- All investigators and staff are GCP trained

# 9.2 DATA MANAGEMENT

Consent form and Case report forms will be filed in a locked filing cabinet in the study coordinator’s locked office.

Data will be double-entered by two research assistants to minimize data entry errors. All electronic data will be stored in a password-protected database. Only individuals listed on the staff form for this study will have access to the database.

# 10 STATISTICAL METHODS

## 10.1 Sample size and power considerations

Using AUC insulin:
By using nQuery Advisor version 6.01, from paper written by Gedik et al (Effects of vitamin D deficiency and repletion on insulin and glucagon secretion in man ^)16^, a sample size of 4 will have 95% power to detect a difference in means of -4.610 (e.g. the First condition mean, µ1, of 9.090 and a Second condition mean, µ2, of 13.700), assuming a standard deviation of differences of 1.200, with a 0.050 two-sided significance level. By adding 40% dropped out patient in case of lost to follow up unplanned pregnancy, the total sample for 2 arms (placebo and treatment group) treatments is 11 patients.


Using HOMA-IR:
By using nQuery Advisor version 6.01, from paper written by Von Hurst et al (Vitamin D supplementation reduces insulin resistance in South Asian women living in New Zealand who are insulin resistant and vitamin D deficient - a randomised, placebo-controlled trial )^31^, a sample size of 9 will have 95% power to detect a difference in means of 0.700 (e.g. the differences between Group 1 mean, µ1, of 0.800 and a Group 2 mean, µ2, of 0.100), assuming that the common standard deviation is 0.384, with a 0.050 two-sided significance level. By adding 40% dropped out patient in case of lost to follow up or unplanned pregnnacy, the total sample for 2 arms (placebo and treatment group) treatments is 26 patients (13 patients for each arms).

Since the study will be using both efficacy analysis, higher sample calculated using HOMA-IR was chosen as reference ie 26 subjects required for study.

Green et al (Vitamin D status and its association with parathyroid hormone concentrations in women of child-bearing age living in Jakarta and Kuala Lumpur)^15^ found 60% of population to have 25 (OH) Vit D of < 50nmol/L. Assuming a similar prevalence exist in our population, we thus expect to screen at least 44 women to recruit 26 subjects for the study.

## 10.2 Randomization

Subjects will be randomly allocated to either Vitamin D3 or Placebo in a 1:1 ratio. Once a patient meets the inclusion and exclusion criteria at the end of screening, she will be randomized through process of central independent randomization with help of CRC hospital Pulau Pinang.

## 10.3 Analysis

### *10.3.1 Analysis Sets*

Analysis will be according to protocol. Two categories of subjects were excluded:

- - Subjects who failed to receive the treatment as intended
  - Subjects who have experienced protocol violation, including missing data on efficacy assessment.

### *10.3.2 Baseline Comparability*

Demographic and baseline characteristic including age, gender, educational attainment, disease status, comorbidity and lab evaluations will be summarized and tabulated by treatment groups.

Continuous variables will be summarized by descriptive statistics, which comprises sample size, mean, median, standard deviation, minimum and maximum. Discrete variables will be summarized by frequencies and percentages (contingency tables).

#

# 11 REFERENCES

1. **Kim C, Newton KM, Knopp RH. Gestational diabetes and the incidence of type 2 diabetes: a systematic review. Diabetes Care 2002;25:1862-8**
2. **Velaiutham S. Glucose intolerance in women with previous gestational diabetes mellitus in penang hospital in relation to clinical and biochemical parameters. Dissertation Submitted for Master of Medicine, University Kebangsaan Malaysia 2007.**
3. Norman AW, Frankel JB, Heldt AM, Grodsky GM: Vitamin D deficiency inhibits pancreatic secretion of insulin. *Science* 209:823– 825, 1980
4. Chiu KC, Chu A, Go VLW, Saad MF. Hypovitaminosis D is associated with insulin resistance and *β* cell Dysfunction. *Am J Clin Nutr* 2004; **79**: 820–825.
5. Scragg R, Sowers M, Bell C. Serum 25-hydroxyvitamin D,diabetes, and ethnicity in the Third National Health and Nutrition Examination Survey. *Diabetes Care* 2004; **27**: 2813–2818.
6. Scragg R, Holdaway I, Singh V, Meltcalf P, Baker J, Dryson E. Serum 25-hydroxyvitamin D3 levels decreased in Impaired glucose tolerance and diabetes mellitus. *Diabetes Res Clin Pract* 1995; **27**: 181–188.
7. Boucher BJ, Mannan N, Noonan K, Hales CN, Evans SJ. Glucose intolerance and impairment of insulin secretion in relation to vitamin D deficiency in east London Asians. *Diabetalogia* 1995; **38**: 1239–1245.
8. Mattila C, Knekt P, Mannisto S, Rissanen H, Laaksonen MA, et al. (2007) Serum 25-hydroxyvitamin D concentration and subsequent risk of type 2 diabetes. Diabetes Care 30: 2569–2570.
9. Forouhi NG, Luan J, Cooper A, Boucher BJ, Wareham NJ (2008) Baseline serum 25-hydroxy vitamin D is predictive of future glycaemic status and insulin resistance: The MRC Ely prospective study 1990-2000. Diabetes 57:2619-2625, 2008
10. Pittas AG, Dawson-Hughes B, Li T, Van Dam RM, Willett WC, Manson JE, Hu B: Vitamin D and calcium intake in relation to type 2 diabetes in women. D*iabetes Care* 29:650–656, 2006
11. R. J. Clifton-Bligh, P. McElduff , A. McElduff et al. Maternal vitamin D deficiency, ethnicity and gestational diabetes. *Diabetic Medicine 2008;* **25:** 678–684
12. *Zhang et al ( 2008)-low vitamind D level at 1^st^ Trimester related to increase risk of GDM –even after adj for age, race, fam hx diabetes ( in a predominantly non asian population)*
13. *Maghbooli Z, Hossein-Nezhad A, Karimi F, Shafaei AR, Larijani B. Correlation between vitamin D3 deficiency and insulin resitance in pregnancy. Diabetes Metab res Rev.2008 Jan-Feb;24(1):27-32*

*(Severe Vit D deficiency (<12.5 nmol/L) more prevalent in GDM than normal pregnancy. Vit D deficiency has strong correlation with Insulin resistance as measured by HOMA- even after correction for age and BMI)*

1. Clemens TL, Henderson SL, Adams JS, Holick MF. Increased skin pigment reduces the capacity of skin to synthesise vitamin D3. *Lancet* 1982; **1**: 74–76.
2. Green T, Skeaff CM, Rockell J et al. Vitamin D status and its association with parathyroid hormone concentrations in women of child-bearing age living in Jakarta and Kuala Lumpur *European Journal of Clinical Nutrition* (2008) **62,** 373–378
3. Gedik O, Akalin S: Effects of vitamin D deficiency and repletion on insulin and glucagon secretion in man. *Diabetologia* 29:142–145, 1986
4. Pittas AG, Harris SS, Stark PC, wson-Hughes B (2007) The effects of calcium and vitamin D supplementation on blood glucose and markers of inflammation in nondiabetic adults. Diabetes Care 30: 980–986.
5. Nilas L, Christiansen C: Treatment with vitamin D or its analogues does not change body weight or blood glucose level in postmenopausal women. *Int J Obes* 8:407– 411, 1984
6. Zofkova I, Stolba P: Effect of calcitriol and trifluoperazine on glucose stimulated B cell function in healthy humans. *Exp Clin Endocrinol* 96:185–191, 1990
7. Fliser D, Stefanski A, Franek E, Fode P, Gudarzi A, Ritz E: No effect of calcitriol on insulin-mediated glucose uptake in healthy subjects. *Eur J Clin Invest* 27:629– 633, 1997
8. Orwoll E, Riddle M, Prince M (1994) Effects of vitamin D on insulin and glucagon secretion in non-insulin-dependent diabetes mellitus. Am J Clin Nutr 59: 1083–1087.
9. Borissova AM, Tankova T, Kirilov G, Dakovska L, Kovacheva R: The effect of vitamin D3 on insulin secretion and peripheral insulin sensitivity in type 2 diabetic patients. *Int J Clin Pract* 57:258– 261, 2003
10. Robert Scragg (2008). Vitamin D and Type 2 Diabetes. Are we ready for a prevention trial? Diabetes 57: 2565-6
11. Holick M. Vitamin D deficiency. N Engl J Med 2007;357:266-81.
12. **Mari A, Pacini G, Murphy E, Ludvik B, Nolan JJ** 2001 A model-based method for assessment of insulin sensitivity from the oral glucose tolerance test. Diabetes Care 24:539–548
13. **Pacini G, Mari A** 2003 Methods for clinical assessment of insulin sensitivity and _-cell function. Best Pract Res Clin Endocrinol Metab 17:305–322
14. **Katz A, Nambi SS, Mather K, Baron AD, Follmann DA, Sullivan G, Quon MJ** 2000 Quantitative insulin sensitivity check index: a simple, accurate method for assessing insulin sensitivity in humans. J Clin Endocrinol Metab 85:2402–2410
15. Matthews D, Hosker J, Rudenski A et al. Homeostatsis model assessment: Insulin resistance and b cell function from fasting plasma glucose and insulin concentration in man. Diabetologia 1985; 28:412-419
16. A. Lapolla, M. G. Dalfra` , G. Mello, Early Detection of Insulin Sensitivity and _-Cell Function with Simple Tests Indicates Future Derangements in Late PregnancyJ Clin Endocrinol Metab, March 2008, 93(3):876–880
17. Paffenbarger RS, Wing AL, Hyde RT. Physical activity as an index of heart attack risk in college alumni. Am J Epidemiol 1978;108:161–75
18. von Hurst PR, [Stonehouse W](http://www.ncbi.nlm.nih.gov/pubmed?term=%22Stonehouse%20W%22%5BAuthor%5D&itool=EntrezSystem2.PEntrez.Pubmed.Pubmed_ResultsPanel.Pubmed_RVAbstract), [Coad J](http://www.ncbi.nlm.nih.gov/pubmed?term=%22Coad%20J%22%5BAuthor%5D&itool=EntrezSystem2.PEntrez.Pubmed.Pubmed_ResultsPanel.Pubmed_RVAbstract) et al.Vitamin D supplementation reduces insulin resistance in South Asian women living in New Zealand who are insulin resistant and vitamin D deficient - a randomised, placebo-controlled trial. Br J Nutr. 2010 Feb;103(4):549-55. Epub 2009 Sep 28.
19. Veith R, Chan PC, MacFarlane GD et al. Efficacy and safety of vitamin D3 intake exceeding the lowest observed adverse effect level. Am J Clin Nutr 2001 Feb; 73(2): 288-94
20. Heaney RP, Davies KM, Chen TC, Holick MF, Barger-Lux MJ: Human serum 25-hydroxycholecalciferol response to extended oral dosing with cholecalciferol. American journal of Clinical Nutrition 2003, 77:204-210
